# Primary Orbital Tuberculosis with Cold Abscess: A Case Report

**DOI:** 10.31729/jnma.8441

**Published:** 2024-02-29

**Authors:** Sanam Dhakal, Sulochana Neupane, Prerna Arjyal Kafle, Badri Prasad Badhu, Santosh Upadhyaya Kafle

**Affiliations:** 1 Department of Ophthalmology, Birat Medical College Teaching Hospital, Tankisinuwari, Morang, Nepal; 2 Department of Ophthalmology, Birat Medical College Teaching Hospital, Tankisinuwari, Morang, Nepal; 3 Department of Clinical Pathology, Birat Medical College Teaching Hospital, Tankisinuwari, Morang, Nepal

**Keywords:** *case reports*, *orbit*, *tuberculosis*

## Abstract

Orbital tuberculosis is a rare form of extrapulmonary tuberculosis involving orbital soft tissue, periosteum, bones and lacrimal glands. This is a case report of a 6-year-old male child who presented with swelling of the right upper eyelid. He had normal visual acuity without signs of diplopia or ophthalmoplegia. The tuberculin skin test was reactive and the computed tomography scan showed peripherally enhancing collection with bony erosion and intracranial extension in the extraconal space of the superolateral right orbit. Orbital exploration was done which showed caseous material. The histological examination revealed necrotizing granulomatous tissue. The caseous material on Ziehl Neelsen staining confirmed acid-fast bacilli causing a tubercular abscess. The child is currently on anti-tubercular therapy planned for 12 months. Orbital tuberculosis might or might not be in association with pulmonary tuberculosis and should always be taken into consideration while dealing with chronic inflammatory orbital disease and an orbital mass.

## INTRODUCTION

Orbital tuberculosis is a rare form of extrapulmonary tuberculosis involving orbital soft tissue, periosteum, bones and lacrimal glands.^1,2^ It can extend to adjacent paranasal sinuses or intracranial cavities.^1^ It has an insidious onset, with patients reporting that symptoms had been present for months to years.^2^ Diagnosis of orbital tuberculosis is based on clinical, radiological, histological and microbiological evidence.^3^ Positive culture remains the gold standard.^4^ The primary modality of treatment of orbital tuberculosis is surgical exploration of orbit with drainage of abscess and antitubercular therapy.^3,5^ Here, we present a case report of tubercular abscess of the orbital wall with bony erosions, as this is one of the rare orbital disorders.

## CASE REPORT

A male child of age 6 years belonging to a lower-middle-class family from Bihar, India presented with complaints of swelling in the superolateral aspect of the right upper eyelid and fever for around 2 months. According to the informant (child's father), the child developed a non-painful, gradually increasing swelling in the right upper eyelid insidiously and was associated with watering of the right eye. There was no overlying skin discolouration or discharge from the swelling.

The patient also had an on/off fever usually occurring during the night, associated with chills plus sweating and didn't subside on taking simple antipyretics. The child was anorexic and had lost weight. There was no history of disturbance in vision, headache, dizziness, loss of consciousness, trauma to eyes and exposure to any allergen, nausea, vomiting, shortness of breath, cough, chest pain, nasal obstruction, abnormal body movements, abdominal pain, loose stool and blood in the stool. The child was completely immunized. There is neither past/present evidence of tuberculosis in the family nor the child had a history of contact with known cases of tuberculosis.

The patient previously received treatment with broad-spectrum antibiotics. The fever subsided but the swelling persisted so the patient visited the hospital. On general physical examination, the child was conscious, alert and well-oriented to time, place and person. He weighed 15 kilograms (< -2 Z score for age). There was no pallor, icterus, cyanosis, clubbing, pedal oedema, dehydration and no lymphadenopathy. Other systemic examinations were normal.

**Figure 1:**
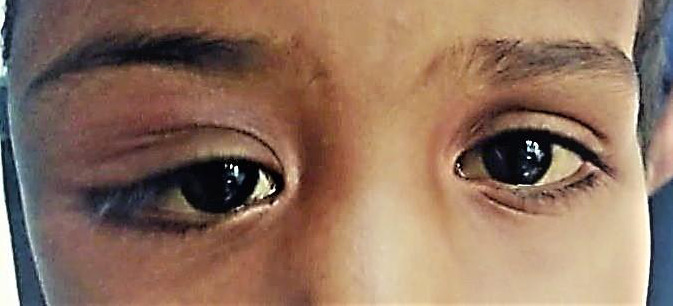
S-shaped deformity in the right upper eyelid margin.

Ocular examination was done with the head being in normal anatomical position (Frankfort's position) with the forehead and bilateral eyebrows being at the same level. On inspection, there was diffuse, irregular swelling involving the lateral aspect of the right upper eyelid and right lateral canthus extending to the ipsilateral temporal region. The right superior eyelid sulcus was obliterated laterally with mild lateral mechanical ptosis giving an S-shaped deformity to the right upper eyelid margin (Figure 1).

The swelling was non-tender, fluctuant and soft in consistency with ill-defined margins. The swelling was approximately 6 cm × 3 cm and depth couldn't be measured out. On Hertel exophthalmometry, there was infero medial non-axial proptosis of the right eye (the right globe was 14 mm and the left 12 mm from the lateral orbital rim). The movement of the right eye was restricted in superolateral, lateral and inferolateral gaze. Movement of the left eye was normal. In both eyes conjunctiva, cornea, iris and other intraocular structures were normal.

**Figure 2:**
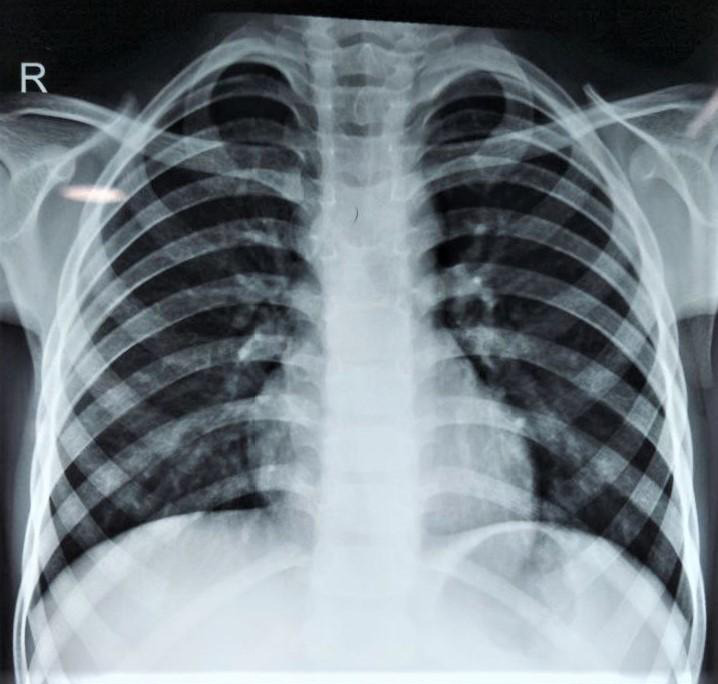
Chest radiograph (Normal study).

Complete blood count (CBC), erythrocyte sedimentation rate (ESR), liver function test (LFT), chest X-ray, abdominal ultrasonography and tuberculin skin test (TST) or Mantoux test were done. There was mild lymphocytosis and ESR in the upper reference level (15 mm/hr) with reactive tuberculin skin test (10 mm). Other tests were normal. The chest radiograph was normal and had no sign of past/recent pulmonary tuberculosis (Figure 2).

**Figure 3:**
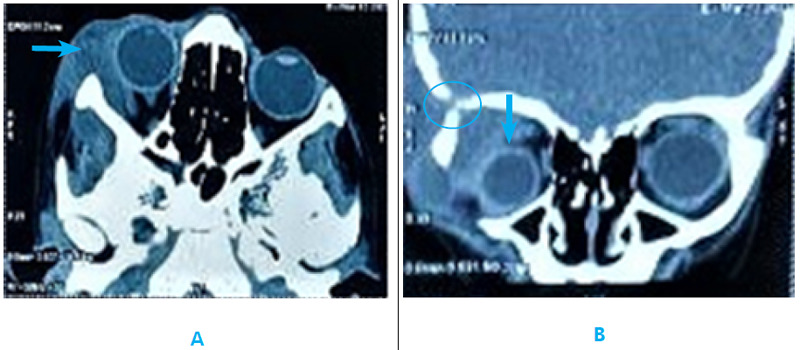
CT orbit (A) axial: right eye medial proptosis with enhancing collection in the right lateral orbital area (arrow) (B) coronal: inferomedial displacement of the right eye (arrow) with superolateral enhancing collection eroding the adjacent orbital bone (circle).

Differential diagnosis of orbital abscess, dacryoadenitis, lymphoma and sarcoidosis was made and a computed tomography (CT) scan of the orbit was done. CT scan of the orbit showed peripherally enhancing collection involving intra and extra-orbital parts in the superolateral aspect of the right orbit with erosion of the superolateral wall and roof of the right orbit with subcutaneous and intracranial extension. These findings were suggestive of infective origin (osteomyelitis with abscess formation) (Figure 3).

Right orbital exploration with drainage of pus and excision of the abscess wall was done. Greyish and creamy caseating material with tissue debris was obtained. Bony defects in a lateral and superolateral orbital wall with a cavity in the temporal region and laterally eroded zygomatic bone were found. Materials were submitted for cytology, Gram-stain, ZN stain, Giemsa stain, KOH-mount (potassium hydroxide) and histopathological examination. Postoperatively, antibiotics (cefixime) and analgesics (paracetamol and ibuprofen) were given both orally and topically.

**Figure 4:**
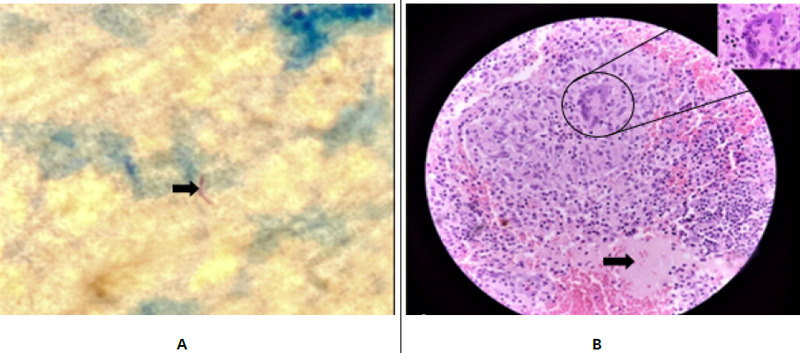
(A) ZN staining showing acid-fast bacillus (B) Hematoxylin and eosin stain (40 X) showing the focus of necrosis (arrow) surrounded by lymphocytes, macrophages, epithelioid and multinucleated giant cells (inside a circle) with foci of haemorrhages (necrotizing granulomatous lesion).

On ZN staining acid fast bacilli was seen (Figure 4). Histopathology showed focal foci of necrosis surrounded by lymphocytes, epithelioid cells, macrophages and multinucleated giant cells with multiple foci of haemorrhages and dilated vascular channels embedded against the background of fibro-collagenous and hemorrhagic stroma (Figure 4b).

These features were of necrotizing granulomatous lesions. Diagnosis of primary orbital tubercular abscess was made and the child was kept on anti-tubercular therapy (ATT) planned for 12 months.

## DISCUSSION

Orbital tuberculosis is a rare form of extrapulmonary tuberculosis. It can be primary or secondary based on origin. Primary orbital tuberculosis represents infection of the orbital/ocular region with localized symptoms but no systemic involvement. Secondary orbital tuberculosis on the other hand represents ocular dissemination of tuberculosis through a hematogenous route from a distant primary site or local extension from nearby structures such as the paranasal sinuses.^1^ Primary tuberculosis is commonly pulmonary in origin, but extrapulmonary sites, like cervical lymphadenopathy or abdominal disease, may be present. Involvement of the lateral wall of the orbit implies a hematogenous source. There has usually been evidence of disseminated tuberculosis in previously reported cases.^5^ In this case the child underwent a chest x-ray to rule out pulmonary foci of tuberculosis. Chest x-ray was normal with no evidence of past/present tuberculosis so other extensive modalities were not involved. So, we couldn't ascertain the origin of tuberculosis in this case.

In a literature review orbital tuberculosis is classified into 5 types, Type (iii), orbital TB with evidence of bony involvement, not categorized as (i), has radiological evidence of bone destruction, osteomyelitis changes and erosion. Clinically proptosis, ophthalmoplegia and diplopia were found in most cases.^3^ This case also shares similar radiological evidence and had proptosis with marked restriction of ocular movement in certain gazes.

Diagnosis of orbital tuberculosis is based on clinical, radiological and histological features of orbital lesion in addition to proven tuberculosis of other parts of the body or demonstration of AFB in discharge/fluid or positive culture.^3^ Polymerase chain reaction (PCR) is an emerging rapid technique for diagnosing both pulmonary and extrapulmonary TB.^3^ Also leukocytosis with elevated ESR is usual blood finding in many cases of orbital tuberculosis.^6^ The initial diagnostic modality in suspected orbital tuberculosis is a CT scan. The radiological findings of CT scan whenever likely, should be further assured with biopsy and histopathology with or without AFB. Positive culture remains the gold standard.^4^ This case also fulfils all those above diagnostic criteria and shares a similar blood picture with the lesional fluid being positive for AFB. In this case, the culture of Mycobacterium tuberculosis was not done. Orbital tuberculosis is one of the paucibacillary forms of extrapulmonary tuberculosis which is usually not infectious but has been synchronously found in smear-positive pulmonary tuberculosis. So, all the patients suspected of orbital tuberculosis should be evaluated for signs of systemic TB (including chest radiograph and sputum microscopy).^3^

In general, the recommendations for the treatment of non-respiratory forms of TB are the same as the respiratory forms with 6-9 months of ATT regimen. In the case of orbital tuberculosis, the recommended modality is the wide surgical removal of the lesion, combined with ATT for 18 months.^3,5^ Surgery in the case of orbital tuberculosis can be diagnostic therapeutic or both.^3^ The standard first-line regimen for drug-sensitive extrapulmonary tuberculosis, according to the WHO guidelines and National Tuberculosis Management Guidelines-2019 Nepal, is a two-month intensive phase with isoniazid (H), rifampicin (R), pyrazinamide (Z) and ethambutol (E) followed by a 10-month continuation phase with isoniazid(H) and rifampicin(R) (2HRZE/10HR).^7^ Following this guideline the child in this case was also started with 2 months of intensive therapy with HRZE and will continue with HR for the next 10 months. On follow-up after 2 months, the swelling had subsided and symptomatic improvement was also noted. The prognosis of orbital tuberculosis with proper treatment is favourable.

Orbital tuberculosis is a rare condition with no specific clinical signs and symptoms except in periostitis form. The mainstay of diagnosis remains the histopathological evaluation. With early diagnosis, appropriate ATT and good compliance towards treatment the prognosis seems good with a low likelihood of infectivity.
